# Supplementary material for: Leveraging Deep Learning Techniques and Integrated Omics Data for Tailored Treatment of Breast Cancer
Source: J Pers Med. 2022 Apr 22;12(5):674. doi: 10.3390/jpm12050674 (PMC9147748; doi:10.3390/jpm12050674)
Supplement: Supplementary file 1 [file jpm-12-00674-s001.zip › jpm-1603541-supplementary.pdf]

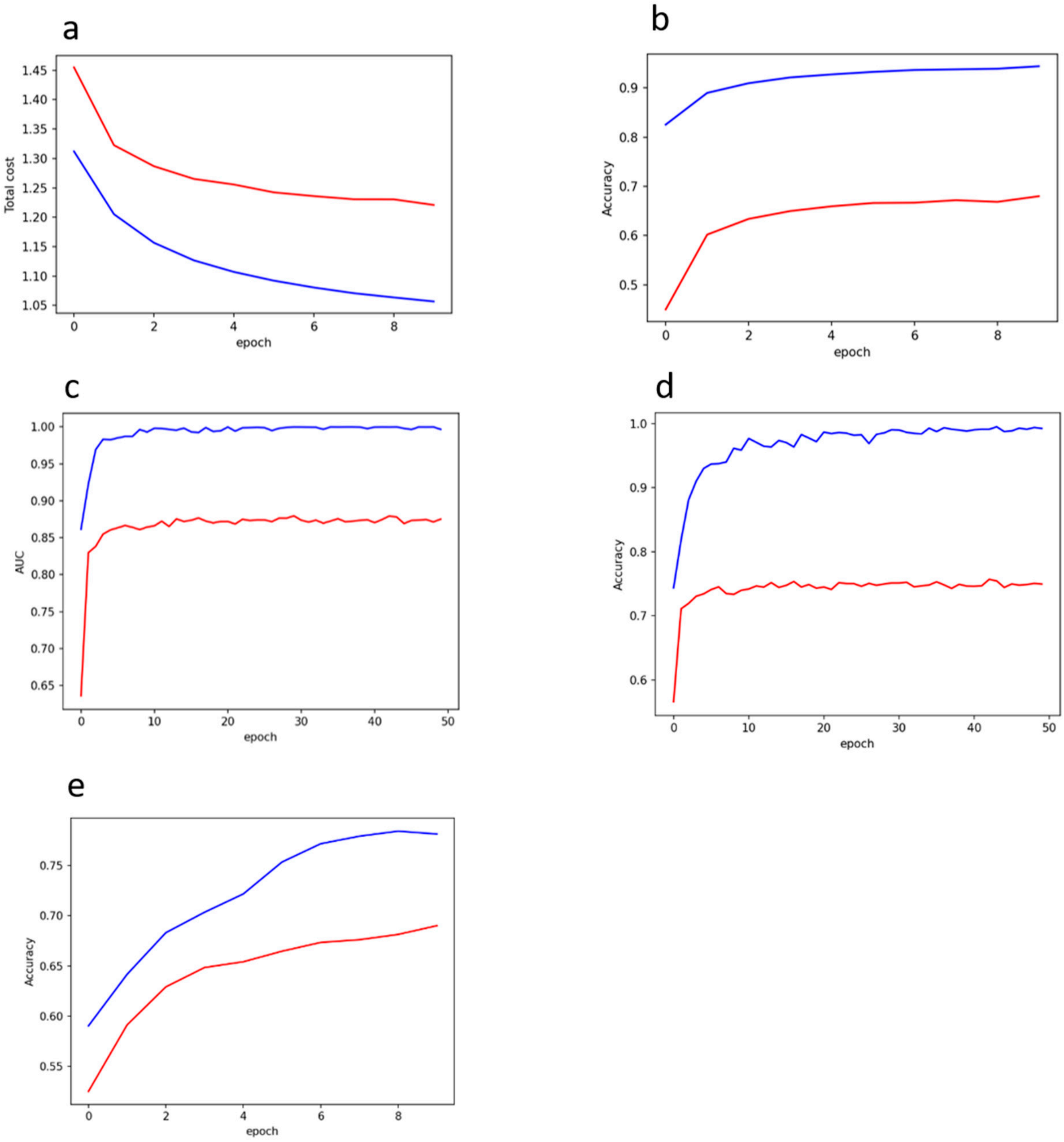

Figure S1. a: Cost of post proposed model using multiomics data and all subtypes. **b**: Accuracy of proposed mode using multiomics data and all subtypes. **c**: Accuracy of the proposed model Lum A vs Her2+ classification. **d**: Accuracy of the proposed model Lum A vs Basal-Like classification. e S1 **e**: Accuracy of the proposed model Lum A vs Lum B Classification. Both worst and best performances are depicted with cross validation.

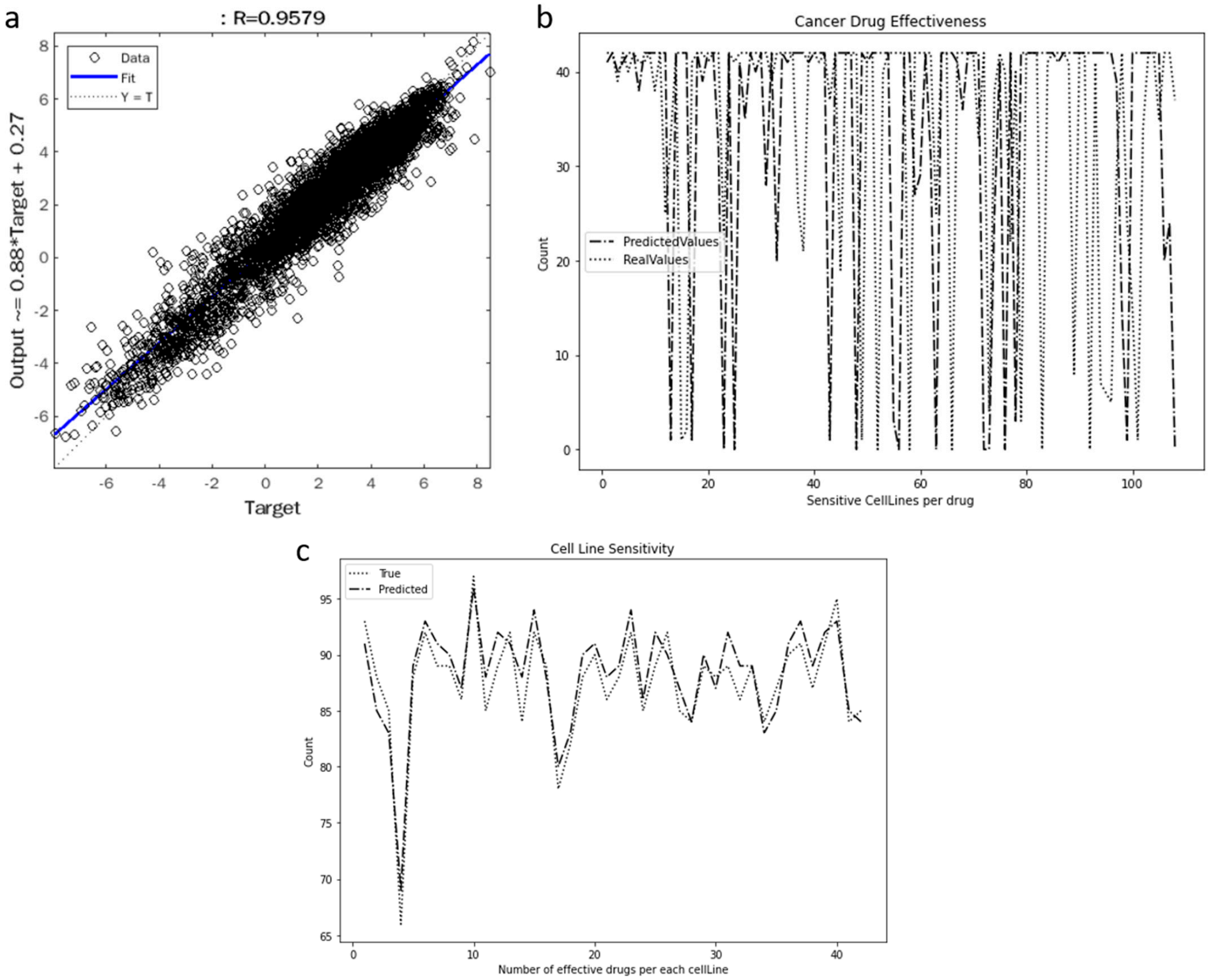

Figure S2. a: Regression values of all drugs combined. **b**: Effectiveness all drugs across all cell lines. **c**: Sensitivity of all cell-lines across all drugs.

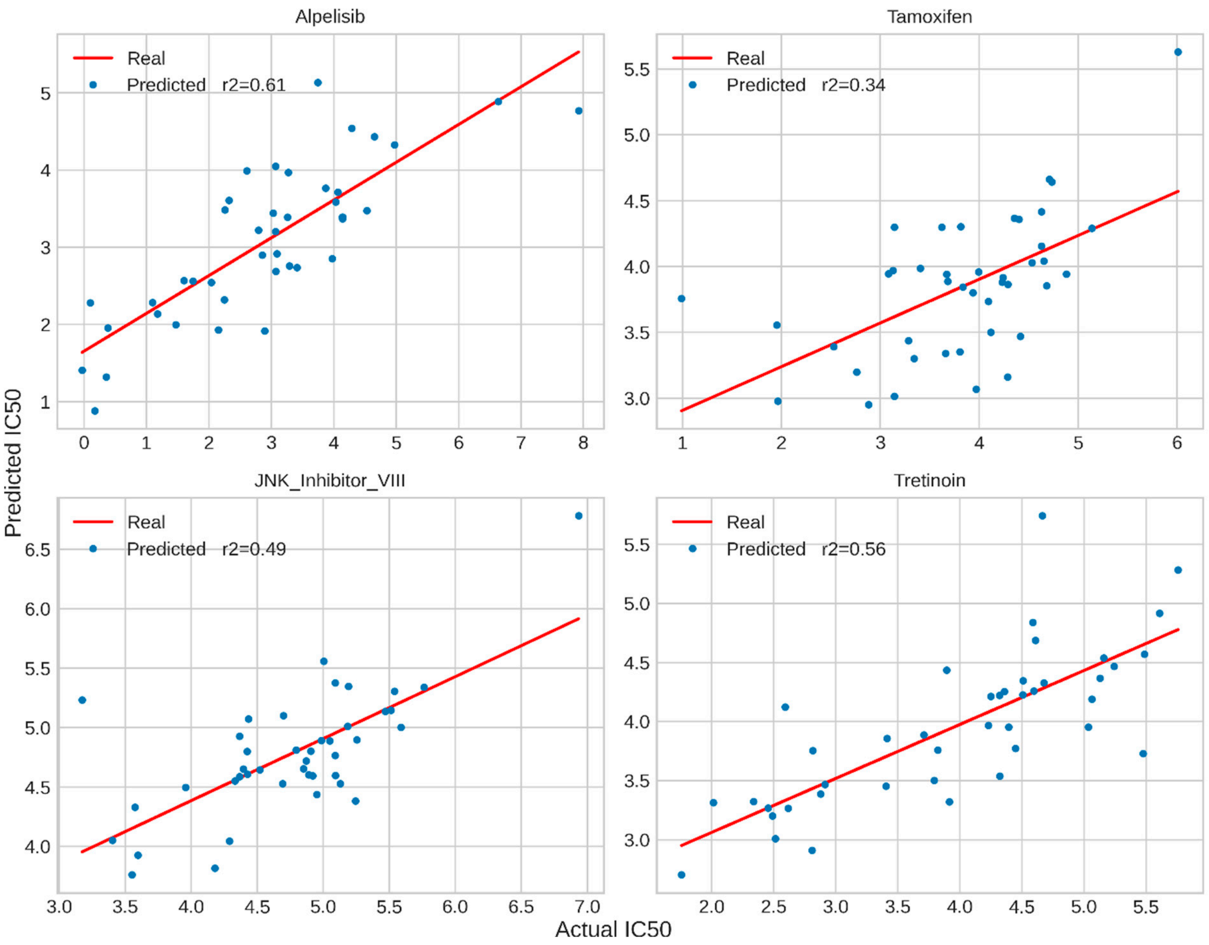

Figure S3. The subplots represent the r2_squared error of the four cancer drugs. The low r2_squared value of each drug is mainly due to outliers.

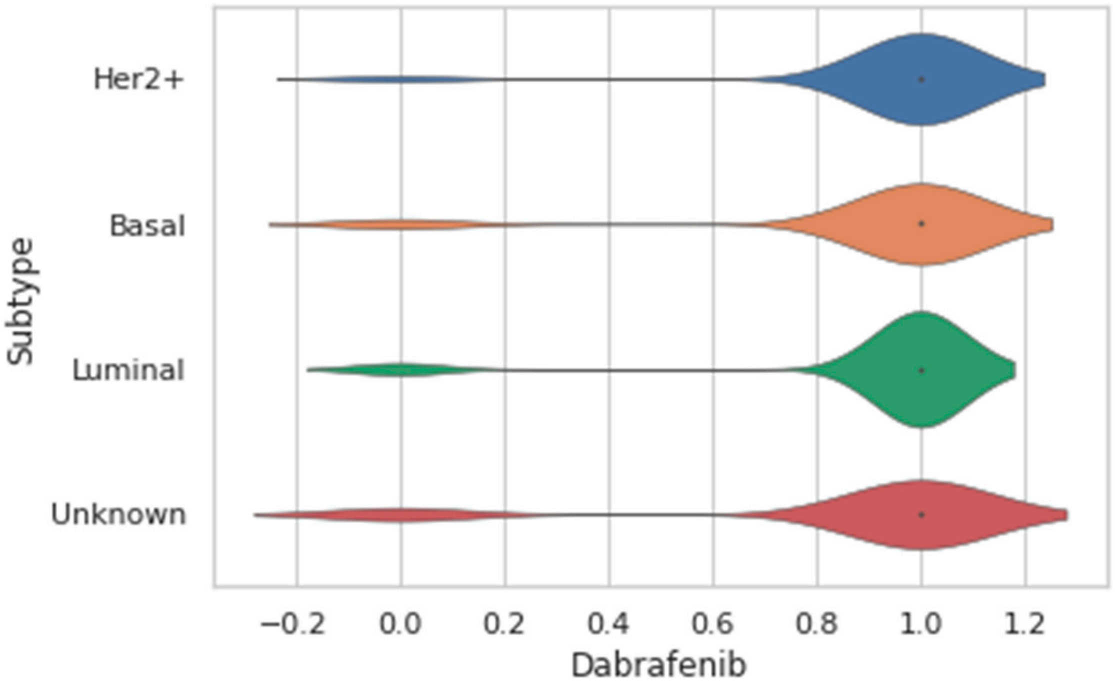

Figure S4. Sensitivity of different subtypes to Dabrafenib.

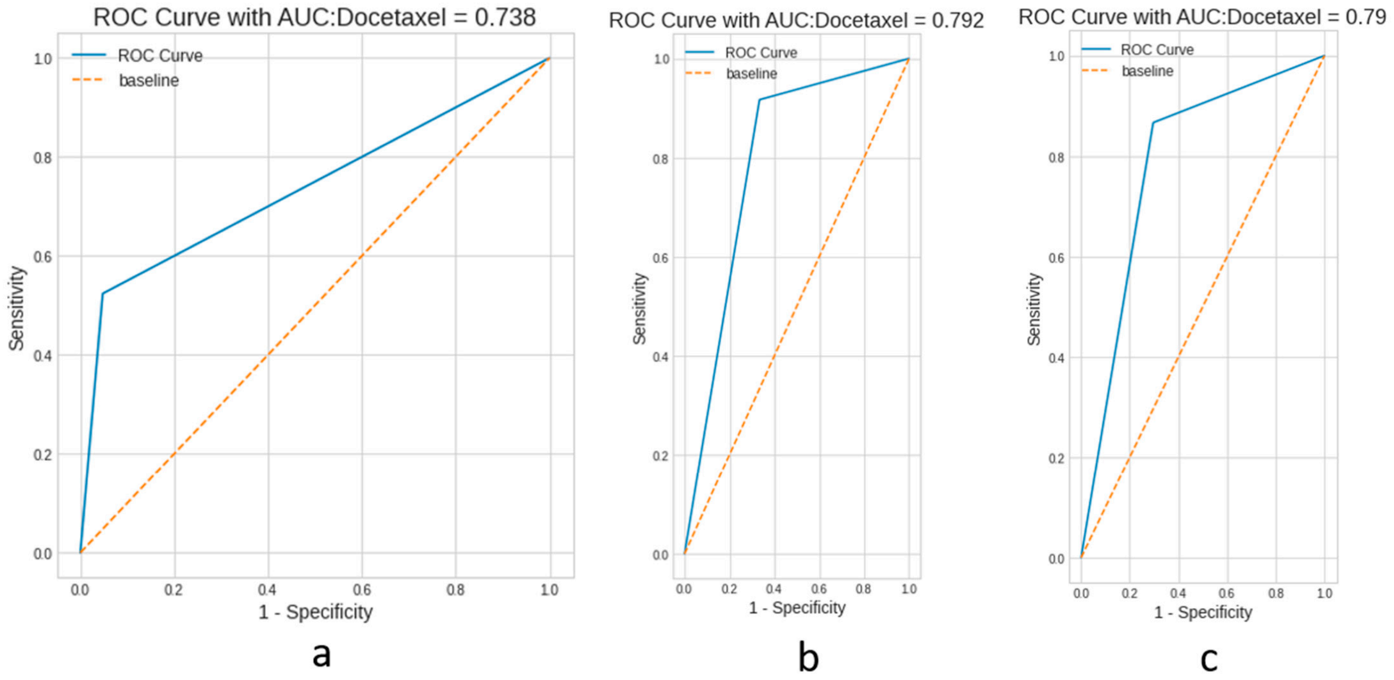

Figure S5. AUC for Docetaxel. **a**: SVM. **b**: Random Forest. **c**: DCNN-DR.

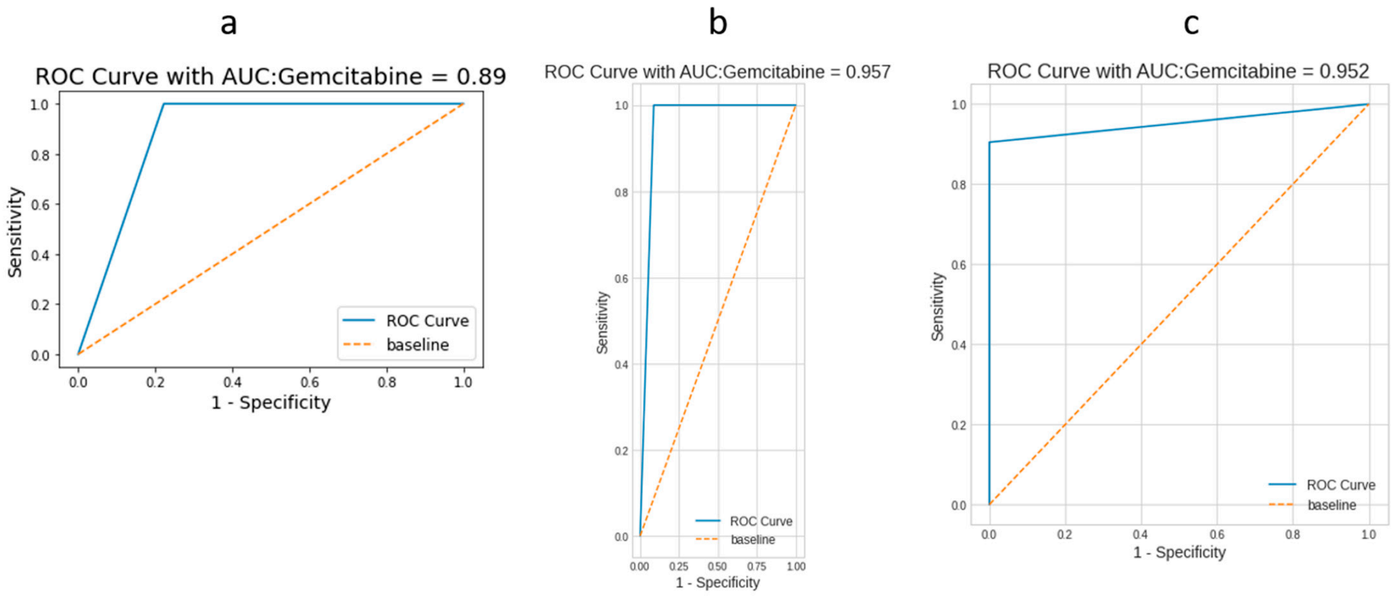

Figure S6. AUC for Gemcitabine. **a**: SVM. **b**: Random Forest. **c**: DCNN-DR.

Table S1. Performance measures of subtype model.

Omics data used	fscore	Accuracy Avg	AUC score	Min Cost
Integrated data auto encoder accuracy	0.96	0.94	0.988	0.96
Single omics protein data all	0.4	0.488	0.68	1.41299
Single omics Methlyation Data	0.86	0.84	0.97	1.078
Single Omics Clinical Data	0.54	0.52	0.75	1.39
Single Omics RNA data	0.9	0.93	0.97	0.97
Single Omics mirna	0.88	0.91	0.975	1.004725

Table S2. Top ranked genes with their p-values after feature engineering.

Features	P values
TP53	5.08×10^{-07}
CDH1	1.34×10^{-04}
EGFR	1.84×10^{-03}
ANKS4B	4.85×10^{-03}
B3GAT1	6.30×10^{-03}
ESR1	6.43×10^{-03}
TMEM90A	6.88×10^{-03}
TRIP10	8.09×10^{-03}
INPP4B	2.53×10^{-02}
SNAP91	3.21×10^{-02}
NDRG1	5.07×10^{-02}
C1orf74	5.49×10^{-02}
BCL2L14	7.11×10^{-02}
DMRT2	1.13×10^{-01}
CFH	1.13×10^{-01}
EN2	1.49×10^{-01}
PGR.1	1.61×10^{-01}
PGR	1.61×10^{-01}

Table S3. PCC and MSE of all the drugs after regression.

Drug number	Drugs	MSE	Pred_values
10	GSK2126458	0.374169	0.95
7	Epothilone_B	0.570993	0.93
11	QL_X_138	0.454059	0.91
14	WZ3105	0.558485	0.91
19	PI_103	0.693053	0.91
30	KIN001_102	0.422952	0.91
29	OSI_027	0.849233	0.91
59	Gemcitabine	1.620999	0.9
64	Bleomycin	1.824436	0.9
51	NPK76_II_72_1	0.491407	0.9
85	AUY922	0.725835	0.9
43	XL_880	0.632381	0.9
94	GSK690693	0.808683	0.89
46	Docetaxel	0.649373	0.89
18	SNX_2112	1.005965	0.89

13	Mitomycin_C	0.500532	0.89
66	Thapsigargin	1.261452	0.89
35	PIK_93	0.45573	0.89
15	CP724714	0.727675	0.88
58	FK866	1.869812	0.88
95	PXD101__Belinostat	0.701246	0.88
63	TPCA_1	0.428334	0.87
69	BMS_345541	0.263834	0.87
38	BX_795	0.436934	0.87
67	AZD7762	0.702587	0.87
42	CAY10603	0.289302	0.87
17	Tipifarnib	0.531419	0.87
55	Afatinib__rescreen_	1.047942	0.87
26	SB_715992	0.853994	0.86
83	PHA_793887	0.737185	0.86
36	JW_7_24_1	0.407229	0.86
31	I_BET	0.553701	0.86
45	CUDC_101	0.841821	0.86
88	ZSTK474	0.632063	0.86
34	HG_6_64_1	0.710582	0.85
41	CX_5461	0.759924	0.85
75	UNC1215	0.097396	0.85
39	STF_62247	0.281229	0.85
68	Genentech_Cpd_10	0.813469	0.84
20	LAQ824	0.642524	0.84
93	KIN001_266	0.481196	0.84
24	AS605240	0.882598	0.84
8	PLX4720__rescreen_	0.271887	0.84
23	GSK429286A	0.234915	0.84
92	YK_4_279	0.451187	0.84
84	KIN001_244	0.293443	0.83
70	Masitinib	0.410133	0.83
73	Trametinib	1.464458	0.83
61	JQ1_1	0.768938	0.83
91	YM201636	0.394204	0.83
100	Taselisib	1.444584	0.83
52	RDEA119	0.771976	0.83
53	XMD14_99	0.216104	0.82
6	VX_11e	0.678445	0.82
28	Ruxolitinib	0.154501	0.82
65	ABT_263	1.186463	0.82
32	Midostaurin	0.788629	0.81
107	Alpelisib	1.045282	0.81
27	TL_1_85	0.547351	0.81
37	MPS_1_IN_1	0.45299	0.81
74	VNLG_124	0.245858	0.81
9	GSK1070916	1.373304	0.81
82	X5_Fluorouracil	0.877591	0.81
98	FTI_277	0.245601	0.81
96	CEP_701	0.690182	0.81
86	SB52334	0.596348	0.81
60	LY317615	1.139537	0.8
1	Dabrafenib	1.067914	0.8
102	Lapatinib	1.041802	0.8
77	AV_951	0.164947	0.8
90	Vinblastine	0.601047	0.8
16	AC220	0.30892	0.8
62	Bicalutamide	0.157783	0.8
50	SN_38	0.991437	0.79

44	UNC0638_1	0.563832	0.79
79	JQ12	1.105395	0.79
22	SGC0946	0.127308	0.79
25	SB590885	0.391449	0.79
57	FR_180204	0.222796	0.78
105	Palbociclib	0.57996	0.78
47	CCT018159	0.513442	0.78
33	Y_39983	0.43126	0.78
78	NSC_207895	0.620491	0.77
56	ATRA	0.50509	0.77
99	Talazoparib	0.693323	0.77
81	EX_527	0.20117	0.76
40	MP470	1.554647	0.74
80	Temozolomide	0.217361	0.74
4	PLX4720	0.578996	0.74
12	NU_7441	0.379083	0.74
2	ABT_888	0.190526	0.73
54	PD_173074	0.599212	0.73
72	Nutlin_3a	0.414614	0.72
71	CCT007093	0.445734	0.72
76	KIN001_055	0.362043	0.71
104	Olaparib	0.534837	0.7
5	JNK_Inhibitor_VIII	0.251564	0.7
106	Ribociclib	0.458063	0.7
101	Dinaciclib	1.32016	0.69
89	NVP_BEZ235	0.392105	0.69
87	Temsirolimus	1.001178	0.68
3	Lenalidomide	0.253377	0.66
97	IOX2	0.268317	0.66
103	Niraparib	0.39966	0.64
49	CH5424802	0.703146	0.63
48	CAL_101	0.511713	0.63
21	JQ1	0.288629	0.61
0	Tamoxifen	0.565824	0.58

Table S4. Performance of classification of drugs as sensitive or resistant.

Sl no	Drugs	FPR	Accuracy	Sensitivity	Specificity
0	Dabrafenib	0.52381	0.6190476	0.76190476	0.47619048
1	Veliparib	0.333333	0.7619048	0.85714286	0.66666667
2	PLX4720	0.285714	0.7380952	0.76190476	0.71428571
3	VX_11e	0.333333	0.7619048	0.85714286	0.66666667
4	Epothilone_B	0.095238	0.8571429	0.80952381	0.9047619
5	PLX4720__rescreen_	0.428571	0.6428571	0.71428571	0.57142857
6	GSK1070916	0.380952	0.7619048	0.9047619	0.61904762
7	Omipalisib	0.095238	0.8571429	0.80952381	0.9047619
8	QL_X_138	0.333333	0.7380952	0.80952381	0.66666667
9	Mitomycin_C	0.047619	0.8571429	0.76190476	0.95238095
10	WZ3105	0.095238	0.7619048	0.61904762	0.9047619
11	CP724714	0.238095	0.8333333	0.9047619	0.76190476
12	Quizartinib	0.333333	0.7857143	0.9047619	0.66666667
13	Tipifarnib	0.238095	0.7380952	0.71428571	0.76190476
14	SNX_2112	0.095238	0.9047619	0.9047619	0.9047619
15	PI_103	0.142857	0.7857143	0.71428571	0.85714286
16	Dacinostat	0.047619	0.8333333	0.71428571	0.95238095
17	SGC0946	0.333333	0.7857143	0.9047619	0.66666667
18	GSK429286A	0.238095	0.8333333	0.9047619	0.76190476
19	AS605240	0.142857	0.7857143	0.71428571	0.85714286

20	SB590885	0.238095	0.8571429	0.95238095	0.76190476
21	Ispinesib Mesylate	0.190476	0.8571429	0.9047619	0.80952381
22	TL_1_85	0.285714	0.7619048	0.80952381	0.71428571
23	Ruxolitinib	0.285714	0.7380952	0.76190476	0.71428571
24	OSI_027	0.095238	0.9047619	0.9047619	0.9047619
25	AKT inhibitor VIII	0.238095	0.8333333	0.9047619	0.76190476
26	I_BET	0.142857	0.8333333	0.80952381	0.85714286
27	Midostaurin	0.142857	0.8809524	0.9047619	0.85714286
28	Y_39983	0.333333	0.7380952	0.80952381	0.66666667
29	HG_6_64_1	0.047619	0.9047619	0.85714286	0.95238095
30	PIK_93	0.095238	0.8571429	0.80952381	0.9047619
31	JW_7_24_1	0.047619	0.9047619	0.85714286	0.95238095
32	MPS_1_IN_1	0.285714	0.7619048	0.80952381	0.71428571
33	BX_795	0.190476	0.8809524	0.95238095	0.80952381
34	STF_62247	0.285714	0.7857143	0.85714286	0.71428571
35	CX_5461	0.333333	0.7857143	0.9047619	0.66666667
36	CAY10603	0.142857	0.6904762	0.52380952	0.85714286
37	Foretinib	0.142857	0.7619048	0.66666667	0.85714286
38	UNC0638_1	0.285714	0.7380952	0.76190476	0.71428571
39	CUDC_101	0.095238	0.7380952	0.57142857	0.9047619
40	Docetaxel	0.047619	0.7380952	0.52380952	0.95238095
41	CCT018159	0.238095	0.7142857	0.66666667	0.76190476
42	SN_38	0.190476	0.7857143	0.76190476	0.80952381
43	NPK76_II_72_1	0.142857	0.7857143	0.71428571	0.85714286
44	Refametinib	0.285714	0.7380952	0.76190476	0.71428571
45	XMD14_99	0.333333	0.7380952	0.80952381	0.66666667
46	Afatinib	0.380952	0.7619048	0.9047619	0.61904762
47	Tretinoin	0.47619	0.7380952	0.95238095	0.52380952
48	FR_180204	0.285714	0.8095238	0.9047619	0.71428571
49	Daporinad	0.190476	0.9047619	1	0.80952381
50	Gemcitabine	0	0.952381	0.9047619	1
51	Enzastaurin	0.095238	0.8333333	0.76190476	0.9047619
52	JQ1_1	0.238095	0.7857143	0.80952381	0.76190476
53	Bicalutamide	0.095238	0.8809524	0.85714286	0.9047619
54	TPCA_1	0.190476	0.8095238	0.80952381	0.80952381
55	Bleomycin	0.142857	0.9047619	0.95238095	0.85714286
56	Navitoclax	0.238095	0.7142857	0.66666667	0.76190476
57	Thapsigargin	0	0.8809524	0.76190476	1
58	AZD7762	0.238095	0.7619048	0.76190476	0.76190476
59	Genentech_Cpd_10	0.190476	0.7380952	0.66666667	0.80952381
60	BMS_345541	0.095238	0.8571429	0.80952381	0.9047619
61	Masitinib	0.047619	0.8095238	0.66666667	0.95238095
62	Trametinib	0.238095	0.8095238	0.85714286	0.76190476
63	VNLG_124	0.190476	0.8571429	0.9047619	0.80952381
64	UNC1215	0.238095	0.8095238	0.85714286	0.76190476
65	Tivozanib	0.238095	0.7857143	0.80952381	0.76190476
66	NSC_207895	0.190476	0.8095238	0.80952381	0.80952381
67	JQ12	0.095238	0.8333333	0.76190476	0.9047619
68	X5_Fluorouracil	0.190476	0.8095238	0.80952381	0.80952381
69	PHA_793887	0.190476	0.8809524	0.95238095	0.80952381
70	KIN001_244	0	0.9047619	0.80952381	1
71	Luminespib	0.142857	0.7619048	0.66666667	0.85714286
72	SB52334	0.333333	0.7380952	0.80952381	0.66666667
73	ZSTK474	0.142857	0.8571429	0.85714286	0.85714286
74	Vinblastine	0.142857	0.8809524	0.9047619	0.85714286
75	YM201636	0.047619	0.8809524	0.80952381	0.95238095

76	YK_4_279	0.142857	0.7619048	0.66666667	0.85714286
77	KIN001_266	0.047619	0.7142857	0.47619048	0.95238095
78	GSK690693	0.142857	0.8333333	0.80952381	0.85714286
79	Belinostat	0.095238	0.7857143	0.66666667	0.9047619
80	Lestaurtinib	0.238095	0.7380952	0.71428571	0.76190476
81	FTI_277	0.142857	0.8095238	0.76190476	0.85714286
82	Talazoparib	0.210526	0.6904762	0.60869565	0.78947368
83	Lapatinib	0.238095	0.6904762	0.61904762	0.76190476
84	Olaparib	0.2	0.6190476	0.45454545	0.8
85	Palbociclib	0.142857	0.7380952	0.61904762	0.85714286
86	Alpelisib	0.25	0.7142857	0.68181818	0.75

Table S5. Predicted drugs for Luminal and Her2 subtypes.

Luminal	Her2+
Lapatinib	Lapatinib
NU_7441	Palbociclib
WZ3105	NU_7441
Quizartinib	WZ3105
Dacinostat	CP724714
SGC0946	Quizartinib
OSI_027	Tipifarnib
STF_62247	Dacinostat
MP470	SGC0946
CCT018159	GSK429286A
Afatinib	OSI_027
Tretinoin	BX_795
FK866	STF_62247
JQ1_1	MP470
Navitoclax	CCT018159
Tivozanib	CH5424802
JQ12	PD_173074
IOX2	Afatinib
	Tretinoin
	FK866
	JQ1_1
	Navitoclax
	UNC1215
	Tivozanib
